# CYP1A2 genotype and acute effects of caffeine on resistance exercise, jumping, and sprinting performance

**DOI:** 10.1186/s12970-020-00349-6

**Published:** 2020-04-15

**Authors:** Jozo Grgic, Craig Pickering, David J. Bishop, Brad J. Schoenfeld, Pavle Mikulic, Zeljko Pedisic

**Affiliations:** 1 Institute for Health and Sport (IHES), Victoria University, Melbourne, Australia; 2 Institute of Coaching and Performance, School of Sport and Wellbeing, University of Central Lancashire, Fylde Road, Preston, PR1 2HE UK; 3 School of Medical and Health Sciences, Edith Cowan University, Joondalup, Australia; 4 Department of Health Sciences, Lehman College, Bronx, USA; 5 Faculty of Kinesiology, University of Zagreb, Zagreb, Croatia

**Keywords:** Supplements, Ergogenic effects, Genetic, Variation

## Abstract

**Background:**

It has been suggested that polymorphisms within CYP1A2 impact inter-individual variation in the response to caffeine. The purpose of this study was to explore the acute effects of caffeine on resistance exercise, jumping, and sprinting performance in a sample of resistance-trained men, and to examine the influence of genetic variation of CYP1A2 (rs762551) on the individual variation in responses to caffeine ingestion.

**Methods:**

Twenty-two men were included as participants (AA homozygotes *n* = 13; C-allele carriers *n* = 9) and were tested after the ingestion of caffeine (3 mg/kg of body mass) and a placebo. Exercise performance was assessed with the following outcomes: (a) movement velocity and power output in the bench press exercise with loads of 25, 50, 75, and 90% of one-repetition maximum (1RM); (b) quality and quantity of performed repetitions in the bench press exercise performed to muscular failure with 85% 1RM; (c) vertical jump height in a countermovement jump test; and (d) power output in a Wingate test.

**Results:**

Compared to placebo, caffeine ingestion enhanced: (a) movement velocity and power output across all loads (effect size [ES]: 0.20–0.61; *p* <  0.05 for all); (b) the quality and quantity of performed repetitions with 85% of 1RM (ES: 0.27–0.85; *p* <  0.001 for all); (c) vertical jump height (ES: 0.15; *p* = 0.017); and (d) power output in the Wingate test (ES: 0.33–0.44; *p* <  0.05 for all). We did not find a significant genotype × caffeine interaction effect (*p*-values ranged from 0.094 to 0.994) in any of the analyzed performance outcomes.

**Conclusions:**

Resistance-trained men may experience acute improvements in resistance exercise, jumping, and sprinting performance following the ingestion of caffeine. The comparisons of the effects of caffeine on exercise performance between individuals with the AA genotype and AC/CC genotypes found no significant differences.

**Trial registration:**

Australian New Zealand Clinical Trials Registry. ID: ACTRN12619000885190.

## Background

Caffeine is one of the most consumed psychoactive stimulants in the world [1]. The effects of caffeine supplementation on exercise performance have received considerable attention in the literature, and the evidence on its ergogenic effects is well-established [1–3]. For example, a recent umbrella review of 21 published meta-analyses reported that caffeine ingestion is acutely ergogenic for aerobic endurance, muscle strength, muscle endurance, power, jumping performance, and exercise speed [3]. Despite these established performance-enhancing effects of caffeine, it is also commonly acknowledged that there is a large degree of variation in response to caffeine supplementation between individuals [4]. Studies that have reported individual participant data suggest that some individuals experience an increase in performance following caffeine ingestion, whereas others do not [4–6]. In order to develop more effective guidelines for caffeine supplementation in sport and exercise settings, the scientific focus has recently been placed on examining and understanding the reasons for the between-individual variation in responses [4, 7].

One potential driver of this individual response is inter-individual genetic variation [4]. The gene CYP1A2 encodes cytochrome P450 1A2, an enzyme responsible for up to 95% of caffeine metabolism [8]. The speed of caffeine metabolism is affected by a single nucleotide polymorphism, rs762551, within this gene [8]. Individuals with the AA genotype at rs762551 are commonly classified as "fast caffeine metabolizers", while C allele carriers (AC/CC genotypes) tend to have a slower clearance of caffeine and are, therefore, commonly classified as "slow caffeine metabolizers" [9]. Significantly greater ergogenic effects of caffeine on aerobic endurance have been reported for individuals with the AA genotype, compared with C allele carriers [6, 10]. However, for high-intensity exercise tasks of a shorter duration, the evidence is less clear.

In a recent study of 19 basketball players, acute ingestion of 3 mg/kg of caffeine produced similar effects on vertical jump performance in individuals with the AA genotype and AC/CC genotypes [11]. These results are in accord with a study that utilized a 30-s Wingate sprint test, while improvement in peak and mean power output was noted following caffeine ingestion, the researchers did not find differences in responses between genotypes [12]. Based on the results of these two studies, it seems variations in the CYP1A2 genotype may not affect the ergogenic effects of caffeine ingestion on high-intensity exercise performance. However, a recent study reported that caffeine ingestion enhances the number of performed repetitions in a resistance exercise session in individuals with the AA genotype but not AC/CC genotypes [13].

Given the conflicting evidence on this topic, the aim of this randomized, double-blind crossover study was to explore the acute effects of caffeine on resistance exercise, jumping, and cycle ergometer sprint performance in a sample of resistance-trained men and the influence of genetic variation of CYP1A2 (rs762551) on the individual variation in responses. We hypothesized that caffeine ingestion would be ergogenic across all exercise tasks and that individuals with the AA genotype would experience greater improvements in exercise performance following caffeine ingestion than those with AC/CC genotypes.

## Methods

### Experimental design

This study employed a double-blind, randomized, crossover design. All participants attended four laboratory sessions. All trials were performed in the morning hours (between 7 am and noon), and at the same time of the day across the sessions for each participant, to ensure that the results were not affected by circadian variation [14]. The trials took place 4 to 7 days apart. The first and second session included familiarization with the exercise protocol (explained in detail in the “Exercise protocol” section). The two main sessions (i.e., caffeine and placebo sessions) were conducted in a randomized and counterbalanced order. The participants were randomly assigned to the two conditions; half of the participants ingested caffeine in the first session and a placebo in the second session, while the other half ingested a placebo in the first session and caffeine in the second session. Participants were asked not to perform any strenuous exercise for at least 24 hours before the main trials. The participants were also asked to keep a food diary for 24 h using “MyFitnessPal” software, and to match their dietary intakes on the days before the two main sessions as much as possible. The participants were required to refrain from caffeine intake after 6 pm on the day prior to the testing [1]. In order to assist with caffeine restriction, we provided the participants with a list of the most common foods and drinks that contain caffeine. The participants arrived at the laboratory following overnight fasting. Caffeine was administered in capsule form, with a dose of 3 mg/kg of body mass (equivalent to the caffeine dose contained in approximately two cups of coffee). The placebo capsule was identical in appearance to the caffeine capsule, but, instead of caffeine, it contained 3 mg/kg of dextrose. The capsules were ingested 60 min before the start of the exercise session [1]. Genotype was determined using a buccal swab. A validated Food Frequency Questionnaire was used to estimate habitual caffeine intake [15]. Prior to the study, the trial was registered in the Australian New Zealand Clinical Trials Registry ID: ACTRN12619000885190.

### Participants

The study involved resistance-trained men as participants. Being resistance-trained was defined in this study as having a minimum of 6 months of resistance training experience with a minimum weekly training frequency of two times on most weeks. All participants were non-smokers. Based on an a priori power analysis done using G*Power software (version 3.1; Germany, Dusseldorf) for repeated-measures Analysis of Variance (ANOVA) (within-between interaction, i.e., in the context of this study genotype × caffeine interaction), with an assumed true effect size *f* of 0.25, the alpha error level of 0.05, and the expected correlation between repeated measures of 0.75, the required sample size to achieve the statistical power of 80% for this study was 18 participants. To factor in possible dropouts, we recruited 22 participants. The exclusion criteria were: (i) prior use of anabolic steroids; and (ii) the existence of any health limitations. Ethical approval for this study was granted by the Victoria University Human Research Ethics Committee (HRE19–019). The remaining data of the project are published elsewhere [16]. Before enrolling in the study, every participant signed an informed consent and filled out a Physical Activity Readiness Questionnaire (PAR-Q). Only participants who responded with ‘No’ to all PAR-Q items were included in the study. In line with previous research [6, 11–13], we combined participants with the AC and CC genotypes into one group (AC/CC group) for the analysis.

#### Exercise protocol

##### One repetition maximum testing

The first two sessions included familiarization with the exercise protocol. These sessions were the same as the main sessions (i.e., placebo and caffeine sessions), with the exception that the first one included one-repetition maximum (1RM) testing in the bench press exercise. For the 1RM test, the participants performed sets of one repetition with progressive increases in load until they reached their estimated 1RM. The load was initially set to 20 kg and subsequently increased by 10 kg increments if the mean concentric velocity of the repetition was 0.4 m/s or higher (as determined by a linear position transducer attached to the barbell). If the mean velocity was lower than 0.4 m/s, the load for the next attempt was adjusted using smaller increases (e.g., 5 kg or 2.5 kg, determined based on consultation with the participants). The participants performed 1RM attempts with progressively increasing loads until the mean velocity was ≤0.2 m/s [17]. When the mean velocity of a successful 1RM attempt reached these values, the load was considered as a valid estimate of the 1RM [17]. Three minutes were allowed between 1RM attempts.

##### Movement velocity and power in the bench press exercise

In the first session, upon determining the 1RM, the participants performed the bench press exercise with loads of 25, 50, 75, and 90% of 1RM [18]. The second, third, and fourth sessions started with the assessment of movement velocity in the bench press exercise with different loads, as the 1RM test was only performed in the first session. The external load was first set at 25% of 1RM and was progressively increased to 90% of 1RM. With each load, the participants performed two sets of one repetition and were instructed to lift the load as fast as possible. The better repetition (in the context of higher movement velocity and power output) was used for the analysis. Each repetition was followed by a 3-min rest interval. During each repetition, a GymAware linear position transducer (GymAware Power Tool, Kinetic Performance Technologies, Canberra, Australia) was attached to the barbell and used to measure mean concentric velocity (m/s), mean power (W), peak concentric velocity (m/s), and peak power (W). Previous research has established that this device has good test-retest reliability for power and velocity outcomes in the bench press [19].

##### Muscle endurance

After the final repetition with 90% of 1RM, participants were provided with 5 min of passive rest. After the rest interval, muscle endurance was assessed with a test that involved performing repetitions to momentary muscle failure with a load corresponding to 85% of 1RM in the bench press exercise, as in the study by Rahimi [13]. Besides the total number of repetitions, we also measured velocity and power output for each repetition using the linear position transducer attached to the barbell. For the purpose of statistical analyses, we compared the total number of repetitions in the placebo and caffeine conditions. We also explored movement velocity and power output of all repetitions by matching the number of repetitions between the placebo and caffeine conditions. For example, if a participant performed eight repetitions following the ingestion of placebo and nine following the ingestion of caffeine, for this part of the analysis, we only considered movement velocity and power output in the first eight repetitions. This approach allowed us to objectively quantify the average quality of the repetitions during the test and examine if caffeine ingestion had an effect on movement velocity and power output when the total number of repetitions was matched.

##### Countermovement jump

After the muscle endurance test, participants rested passively for 3 minutes and then performed 1 minute of light running, followed by 10 bodyweight squats, in order to warm-up for the countermovement jump (CMJ). The participants performed a CMJ on a force platform (400S Isotronic Fitness Technology, Skye, South Australia, Australia). The CMJ was performed without an arm swing. The participants started CMJ testing from an upright standing position on the force platform. The participants positioned themselves in the starting position and then received commands from the software displayed on a computer screen that was in front of the platform. The software counted down, “3, 2, 1” and provided “Set” and “Go” commands. After the “Go” command, the participants had 5 seconds to complete the jump. From the starting position, the participants performed a downward countermovement (i.e., a fast knee flexion) where their lowest position was a semi-squat position (knee ~ 90° and trunk/hips in a flexed position) [20]. Immediately after reaching this point, the participants performed an "explosive" extension of the legs [20]. The participants were given instructions to jump as quickly and "explosively" as possible to achieve maximal vertical jump height [20]. The participants had one warm-up jump and three official attempts. Each attempt was followed by 1 minute of rest. For the analysis, the best jump from three official attempts was used. The outcome in the CMJ test was vertical jump height, determined by an algorithm based on the flight time.

##### Wingate test

After the CMJ test, the participants were provided another 3 minutes of passive rest before starting the Wingate test. The Wingate test was performed using a Lode Excalibur Sport Cycle Ergometer (The Netherlands, Groningen). Individual setup of the cycle ergometer; namely, saddle and handlebar height and length, was determined in the first session and was maintained throughout all subsequent trials. The Wingate test started with a 5-min warm-up (100 W at 60–80 rpm) [21]. After the warm-up, participants performed a 30-s "all-out" sprint while the resistance placed on the flywheel remained constant at 0.75 Nm/kg. The participants remained seated during the 30-s sprint. During the test, peak power, mean power, and minimum power were recorded using the Lode Ergometry Manager 10 software. Peak power was defined as the greatest power value recorded during the 30-s; mean power was the arithmetic mean of power during the test, and minimum power was the lowest power recorded during the sprint.

### Side effects

Side effects of caffeine and placebo supplementation were evaluated at two time points: (1) immediately after the completion of the testing sessions; and (2) in the following mornings, upon waking. The participants responded to an 8-item survey regarding the incidence of side effects (“yes/no” response scale). This survey was also used to examine side effects in previous research that explored effects of caffeine on exercise performance [20, 22, 23].

### Assessment of blinding

Both in the caffeine and the placebo trials, before and after the exercise session, participants responded to the following question: “Which supplement do you think you have ingested?” [24]. The question had three possible responses: (a) “caffeine”, (b) “placebo” and (c) “I do not know” [24]. In case participants respond with “a” or “b”, they were required to state the reason for choosing their response.

### Genetic testing

The participants underwent genetic testing using a commercially available testing kit from DNAfit Life Sciences (London, UK), as in other studies [25]. Samples were collected using buccal swab devices, with OCR-100 kits by DNAGenotek (Ottawa, Canada). The participants were required to avoiding eating or drinking for at least 60 min prior to the sample collection. All samples were collected according to the manufacturer guidelines. The samples were sent to IDna Genetics Laboratory (Norwich, UK), where the analysis was performed. DNA was extracted and purified using the Isohelix Buccalyse DNA extraction kit BEK-50 (Cell Projects Ltd., Kent, UK), and amplified through polymerase chain reaction (PCR) on an ABI 7900 real-time thermocycler (Applied Biosystem, Waltham, USA). The samples were analyzed for the CYP1A2 rs762551 single-nucleotide polymorphism. This analysis was performed after the exercise performance data collection; thus, the researchers and participants were blinded to genotype variations of the cohort until the data collection process was finalized.

#### Statistical analysis

One-way ANOVA was used to test the differences between genotype groups in age, body mass, height, 1RM, and habitual caffeine intake. We used a two-way, repeated-measures ANOVA to test genotype (AA genotype vs. AC/CC genotypes) × caffeine (placebo vs. caffeine) interaction effect on performance data, separately for each performance variable. In the absence of significant genotype × caffeine interaction effects, we conducted no stratified analyses of the effects of caffeine by genotype groups. Relative effect sizes (ES) were calculated as Hedge’s *g* for repeated measures and presented together with their respective 95% confidence intervals (95% CIs). ESs of < 0.20, 0.20 to 0.49, 0.50 to 0.79, and ≥ 0.80 were considered to represent trivial, small, moderate, and large effects, respectively. McNemar’s test was used in the comparison of the incidence of side effects between the placebo and caffeine conditions. The blinding data were summarized using the Bang’s Blinding Index [26]. The values in this index range from *−* 1.0 (denoting opposite guessing) to 1.0 (denoting complete unblinding) [26]. For this study, we reported the data from this index as a percentage of individuals who identified the correct treatment condition beyond chance [19, 26]. All analyses were performed using the Statistica software (version 13.4.0.14; TIBCO Software Inc., Palo Alto, CA, USA). The significance level was set at *p* <  0.05.

## Results

### Study participants

All participants completed all testing procedures and were included in the final analysis. Of the whole sample, 13, 7, and 2 participants were categorized as having the AA, AC, or CC genotype, respectively. The participants’ characteristics are presented in Table 1. There were no significant differences between the genotype groups for age, body mass, height, 1RM, or habitual caffeine intake.

**Table 1:** Characteristics of the participants

Variable	AA group (*n* = 13)	AC/CC group (*n* = 9)	*p*-values from one-way ANOVA
Age (years)	27.0 ± 5.6	29.8 ± 3.6	0.205
Body mass (kg)	78.2 ± 6.5	80.9 ± 14.8	0.559
Height (cm)	182.2 ± 5.5	183.2 ± 5.7	0.658
1RM in the bench press (normalized per body mass)	1.1 ± 0.1	1.2 ± 0.2	0.240
Habitual caffeine intake (mg/day)	133 ± 123	117 ± 68	0.286

Data reported as mean ± standard deviation; *1RM* one repetition maximum; habitual caffeine intake was estimated using a Food Frequency Questionnaire

### Movement velocity and power output in the bench press exercise

We did not find a significant main effect for genotype (*p* > 0.05 for all) or a genotype × caffeine interaction effect for any of the 16 analyzed variables for movement velocity and power output in the bench press exercise (mean power, mean velocity, peak power, and peak velocity at 25, 50, 75, and 90% 1RM; Table 2). For all variables, except peak power output at 50% 1RM, there was a significant main effect favoring caffeine (*p* <  0.05). The ESs, favoring caffeine conditions in all outcomes, ranged from 0.20 to 0.29 for all outcomes recorded at 25% 1RM, from 0.21 to 0.23 for all outcomes at 50% 1RM, from 0.31 to 0.50 for all outcomes at 75% 1RM, and from 0.57 to 0.61 for outcomes at 90% 1RM.

**Table 2:** Effects of caffeine on resistance exercise, jumping, and sprinting performance: results from the two-way, repeated-measures ANOVA

Variable	AA genotype (placebo)	AA genotype (caffeine)	AC/CC genotypes (placebo)	AC/CC genotypes (caffeine)	Main effect for genotype ***p***-value	Main effect for caffeine ***p***-value	Genotype × caffeine interaction effect ***p***-value	Effect size for condition and its 95% CI
*Movement velocity and power in the bench press with different loads*								
MP at 25% 1RM (W)	1892 ± 299	2012 ± 325	2152 ± 501	2279 ± 517	0.139	0.001	0.918	0.29 (0.12, 0.46)
MV at 25% 1RM (m/s)	1.41 ± 0.12	1.44 ± 0.14	1.46 ± 0.16	1.49 ± 0.15	0.411	0.035	0.566	0.20 (0.02, 0.39)
PP at 25% 1RM (W)	3287 ± 374	3409 ± 384	3598 ± 688	3703 ± 804	0.215	0.033	0.868	0.20 (0.03, 0.37)
PV at 25% 1RM (m/s)	2.21 ± 0.18	2.27 ± 0.18	2.31 ± 0.20	2.35 ± 0.17	0.244	0.008	0.806	0.26 (0.07, 0.46)
MP at 50% 1RM (W)	1182 ± 145	1217 ± 154	1279 ± 214	1333 ± 249	0.196	0.008	0.545	0.22 (0.06, 0.39)
MV at 50% 1RM (m/s)	0.94 ± 0.08	0.97 ± 0.08	0.96 ± 0.11	0.98 ± 0.10	0.711	0.019	0.955	0.21 (0.02, 0.42)
PP at 50% 1RM (W)	1979 ± 201	2036 ± 220	2122 ± 394	2203 ± 406	0.228	0.090	0.753	0.21 (− 0.03, 0.46)
PV at 50% 1RM (m/s)	1.41 ± 0.09	1.43 ± 0.09	1.44 ± 0.18	1.48 ± 0.16	0.468	0.031	0.489	0.23 (0.03, 0.45)
MP at 75% 1RM (W)	789 ± 144	838 ± 151	849 ± 148	928 ± 198	0.281	< 0.001	0.229	0.36 (0.19, 0.56)
MV at 75% 1RM (m/s)	0.56 ± 0.07	0.60 ± 0.07	0.58 ± 0.10	0.63 ± 0.10	0.618	< 0.001	0.514	0.48 (0.27, 0.72)
PP at 75% 1RM (W)	1210 ± 238	1289 ± 233	1369 ± 207	1453 ± 293	0.128	0.007	0.940	0.31 (0.10, 0.54)
PV at 75% 1RM (m/s)	0.80 ± 0.12	0.88 ± 0.09	0.86 ± 0.17	0.91 ± 0.17	0.433	< 0.001	0.243	0.50 (0.26, 0.77)
MP at 90% 1RM (W)	501 ± 128	582 ± 132	588 ± 109	675 ± 143	0.103	< 0.001	0.850	0.61 (0.31, 0.93)
MV at 90% 1RM (m/s)	0.33 ± 0.06	0.38 ± 0.07	0.38 ± 0.12	0.43 ± 0.09	0.182	< 0.001	0.909	0.57 (0.28, 0.89)
PP at 90% 1RM (W)	821 ± 225	970 ± 231	994 ± 301	1165 ± 308	0.099	< 0.001	0.789	0.57 (0.25, 0.91)
PV at 90% 1RM (m/s)	0.50 ± 0,09	0.59 ± 0.11	0.59 ± 0.18	0.67 ± 0.13	0.117	< 0.001	0.966	0.59 (0.27, 0.95)
*Muscle endurance test*								
Maximum repetitions at 85% 1RM	6.8 ± 2.3	8.2 ± 2.2	7.8 ± 2.4	8.8 ± 2.2	0.397	< 0.001	0.454	0.53 (0.27, 0.81)
MP matched for repetitions (W)	376 ± 86	449 ± 96	476 ± 122	531 ± 159	0.074	< 0.001	0.406	0.53 (0.31, 0.79)
MV matched for repetitions (m/s)	0.25 ± 0.04	0.30 ± 0.04	0.30 ± 0.05	0.33 ± 0.04	0.034	< 0.001	0.094	0.85 (0.50, 1.25)
PP matched for repetitions (W)	607 ± 178	674 ± 187	741 ± 297	808 ± 300	0.201	< 0.001	0.994	0.27 (0.14, 0.41)
PV matched for repetitions (m/s)	0.38 ± 0.06	0.43 ± 0.05	0.44 ± 0.09	0.48 ± 0.08	0.108	< 0.001	0.198	0.51 (0.28, 0.77)
*CMJ*								
CMJ vertical jump height (cm)	34.8 ± 6.2	35.6 ± 5.9	36.6 ± 5.2	37.6 ± 5.4	0.447	0.017	0.752	0.15 (0.03, 0.28)
*Wingate*								
PP in the Wingate test (W)	874 ± 208	943 ± 197	864 ± 273	954 ± 260	0.998	< 0.001	0.542	0.33 (0.16, 0.52)
MP in the Wingate test (W)	583 ± 77	614 ± 67	606 ± 120	646 ± 132	0.517	< 0.001	0.583	0.35 (0.20, 0.52)
MinP in the Wingate test (Watts)	338 ± 108	372 ± 79	350 ± 109	414 ± 114	0.505	0.011	0.396	0.44 (0.09, 0.81)

*MP* mean power, *MV* mean velocity, *PP* peak power, *PV* peak velocity, *1RM* one repetition maximum, *MinP* minimum power, *CMJ* countermovement jump, *CI* confidence interval

### Muscle endurance

For the maximum number of repetitions in the bench press exercise with 85% 1RM, we did not find a significant main effect for genotype (*p* = 0.397) or a genotype × caffeine interaction effect (*p* = 0.454), while there was a significant main effect favoring caffeine (*p* <  0.001; ES = 0.53). For peak velocity, mean power output, and peak power output (matched for repetitions between placebo and caffeine conditions), we did not find a significant main effect for genotype (*p* > 0.05 for all) or a genotype × caffeine interaction effect (*p* > 0.05 for all), while there was a significant main effect favoring caffeine in all three variables (*p* <  0.001 for all). The ESs ranged from 0.27 to 0.53. For mean velocity, there was a significant main effect for genotype (*p* = 0.034), with the AC/CC genotypes producing greater movement velocity than the AA genotype, and a significant main effect favoring caffeine (*p* <  0.001; ES = 0.85), while we found no significant genotype × caffeine interaction effect (*p* = 0.094).

### Countermovement jump

For vertical jump height in the CMJ test, we did not find a significant main effect for genotype (*p* = 0.447) or a genotype × caffeine interaction effect (*p* = 0.752), while there was a significant main effect favoring caffeine (*p* = 0.017; ES = 0.15).

### Wingate test

For peak power in the Wingate test, we did not find a significant main effect for genotype (*p* = 0.998) or a genotype × caffeine interaction effect (*p* = 0.542), while there was a significant main effect favoring caffeine (*p* < 0.001; ES = 0.33). For mean power in the Wingate test, we did not find a significant main effect for genotype (*p* = 0.517) or a genotype × caffeine interaction effect (*p* = 0.583), while there was a significant main effect favoring caffeine (*p* < 0.001; ES = 0.35). For minimum power in the Wingate test, we did not find a significant main effect for genotype (*p* = 0.505) or a genotype × caffeine interaction effect (*p* = 0.396), while there was a significant effect favoring caffeine (*p* = 0.011; ES = 0.44).

### Side effects

In the responses recorded immediately post-exercise, we found a significant difference between the placebo and caffeine conditions only in items “Increased vigor/activeness” and “Perception of improved performance” in the AC/CC genotypes (Table 3). In the responses 24-h after capsule ingestion, we did not find any significant differences in the incidence of side effects between the placebo and caffeine conditions.

**Table 3:** Perceived side effects based on questionnaires completed immediately after the testing session and the following morning

Variable	AA group – placebo	AA group – caffeine	AC/CC group – placebo	AC/CC group – caffeine
**Immediately after testing session**				
Muscle soreness	46%	23%	0%	0%
Increased urine production	0%	23%	0%	11%
Tachycardia and heart palpitations	8%	8%	0%	0%
Increased anxiety	0%	23%	0%	0%
Headache	8%	8%	11%	11%
Abdominal/gut discomfort	0%	0%	0%	0%
Increased vigor/activeness	23%	62%	0%^a^	67%^a^
Perception of improved performance	15%	62%	11%^a^	100%^a^
**The following morning**				
Muscle soreness	23%	8%	0%	22%
Increased urine production	8%	0%	0%	11%
Tachycardia and heart palpitations	0%	0%	0%	0%
Increased anxiety	0%	0%	0%	0%
Headache	8%	8%	22%	0%
Abdominal/gut discomfort	0%	0%	0%	0%
Insomnia	8%	0%	0%	11%
Increased vigor/activeness	0%	0%	0%	33%

^a^Significant difference between the placebo and caffeine conditions within a group

### Assessment of blinding – AA genotype

Before starting the exercise session, in the placebo and caffeine conditions, respectively, 62% and 54% of the participants with the AA genotype correctly guessed the treatment identity beyond chance. After exercise, in the placebo and caffeine conditions, respectively, 85% and 69% of the participants with the AA genotype correctly guessed the treatment identity beyond chance.

### Assessment of blinding – AC/CC genotypes

Before starting the exercise session, in both the placebo and caffeine conditions, 55% of the participants with the AC/CC genotypes correctly guessed the treatment identity beyond chance. After exercise, in the placebo and caffeine conditions, respectively, 44% and 78% of the participants with the AC/CC genotypes correctly guessed the treatment identity beyond chance, respectively.

## Discussion

The results of the present study demonstrate that the acute ingestion of a moderate dose of caffeine (3 mg/kg) may produce significant improvements in: (a) movement velocity and power output in the bench press using loads ranging from 25 to 90% of 1RM; (b) maximum number of repetitions performed to momentary muscle failure in the bench press exercise, as well as the average quality (i.e., higher movement velocity and power output) of the performed repetitions; (c) vertical jump height; and (d) peak, mean, and minimum power in the 30-s Wingate test. No significant differences in the effects of caffeine were found between the individuals with the AA genotype and the individuals with the AC/CC genotypes in any of the performance tests used in the present study.

### Effects of caffeine on exercise performance

In the bench press exercise, caffeine ingestion enhanced peak and mean velocity and consequently, mean and peak power, when exercising with low, moderate, and high loads. These results are generally in line with previous findings [18, 20, 22]. One of the early studies [18] conducted on this topic reported that high doses of caffeine (9 mg/kg) are required for acute increases in movement velocity when exercising with very high loads (90% 1RM). However, our results suggest that a dose of 3 mg/kg is effective for enhancing velocity across a wide range of external loads, suggesting that very high doses might not be needed. This is especially relevant to highlight as the ESs in our study are very similar to those reported for the bench press exercise by Pallarés et al. [18].

A recent meta-analysis found that caffeine ingestion enhances mean and peak movement velocity in resistance exercise [27]. The researchers also noted that the effects of caffeine on mean velocity (ES = 0.80) were higher than those for peak velocity (ES = 0.41) [27]. However, the studies included in that meta-analysis assessed either mean or peak velocity; that is, no studies included in the meta-analysis measured both outcomes in the same group of participants [27]. In the present study, we found that the ESs were very similar for both mean and peak velocity, and this was a constant finding across all the employed loads (i.e., 25 to 90% of 1RM).

The muscle endurance test used in this study further confirmed that caffeine ingestion is ergogenic for this fitness component in resistance-trained men. This study adds to the body of evidence showing improvements in muscle endurance following caffeine ingestion [28–32]. However, a more novel finding is that caffeine is ergogenic for power and velocity outputs when the number of repetitions between the caffeine and placebo conditions is matched. Specifically, when matching the number of repetitions between conditions, we found that the effects of caffeine, as compared to placebo, amounted to 0.27 for peak power, 0.51 for peak velocity, 0.53 for mean power, and 0.85 for mean velocity. Several studies that explored the effects of caffeine on muscle endurance did not find a difference in the number of performed repetitions between the caffeine and placebo conditions [13, 33, 34]. However, as we demonstrated in the present study, even with an equal number of repetitions between conditions, caffeine might have still produced considerable improvements in the quality of the performed repetitions, that is, greater movement velocity and consequently, greater power output (which was not tested in the aforementioned studies). As compared to placebo, caffeine ingestion most commonly produced moderate improvements in the number of performed repetitions (generally one to three additional repetitions) [28, 31]. We propose that in some contexts, improvements in the overall quality of the performed repetitions may be more important for training adaptations than simply performing a greater number of repetitions. This hypothesis is in line with recent findings that training at a velocity loss of 20% produced greater improvement in CMJ performance than training at a 40% velocity loss [35]. Improvements in squat strength were similar for both training conditions, even though the group that trained with a velocity loss of 20% performed 40% fewer repetitions.

Caffeine ingestion resulted in increased vertical jump height in the CMJ. The ES magnitude of 0.15 observed in this study is very similar to the pooled ES of 0.17 reported in a recent meta-analysis of 10 studies [36]. This result, therefore, confirms that caffeine ingestion may have a relatively small performance-enhancing effect on vertical jump height [36–38]. The acute improvement in vertical jump height following caffeine ingestion is comparable to the improvement in jump height found as a result of 4 weeks of plyometric training [39, 40]. Even though the improvement in performance was relatively small (approximately 1 cm), it might still be practically meaningful in sports where jump height directly impacts athletic outcomes.

In the Wingate test, we found a significant ergogenic effect of caffeine on peak, mean, and minimum power. These results are in line with the findings of a recent meta-analysis that reported ergogenic effects of caffeine on mean and peak power in the ES magnitude of 0.18 and 0.27, respectively [41]. Of the 16 studies included in the meta-analysis [41], 12 studies used caffeine doses of 5 or 6 mg/kg. Therefore, it could be argued that the findings of the meta-analysis should primarily be generalized to these doses of caffeine. In the present study, we found that even a lower dose of caffeine (namely, 3 mg/kg), increases performance in this test and that the ES is very similar to that reported by studies using higher caffeine doses [41].

### The influence of the CYP1A2 genotype

We did not find significant genotype × caffeine interaction effects in any of the analyzed performance variables. It might be that the effects of caffeine ingestion are similar between different CYP1A2 genotypes, at least for the performance tests used in the present study. The results reported herein are generally in line with the current body of evidence. Two studies [11, 12] that explored the effects of caffeine on jumping and Wingate test performance reported similar improvements in these outcomes following the ingestion of 3 mg/kg of caffeine in groups of participants with the AA and AC/CC genotypes. However, a recent study [13] that used a resistance exercise protocol, found that caffeine is ergogenic only for individuals with the AA genotype. On average, individuals with the AA genotype were able to complete one more repetition with the consumption of caffeine, as compared to placebo, whereas the number of repetitions was the same in the placebo and caffeine conditions among those with the AC/CC genotypes. The main methodological difference between the current studies exploring this topic was the dose of caffeine administered to the participants. Specifically, we and two other studies that reported similar results utilized 3 mg/kg of caffeine. We opted to utilize a lower dose of caffeine as higher doses of caffeine do not seem to produce greater increases in performance [28]. In the study by Rahimi [13], the dose was considerably higher (i.e., 6 mg/kg). It might be that the differences in responses between genotypes become apparent only at higher doses of caffeine. Future dose-response studies might consider exploring this hypothesis further. The effectiveness of the blinding was not explored by Rahimi [13] thus limiting the comparison of the results in this aspect of the study design.

Even though Rahimi [13] reported that caffeine ingestion is ergogenic for AA but not AC/CC genotypes in resistance exercise, the main outcome of that study was the number of performed repetitions in 4 different resistance exercises with 85% 1RM, which can be considered as a somewhat crude test of performance. As mentioned previously, we demonstrated that even when matched for the number of repetitions, caffeine, as compared to placebo, increases the average movement velocity and power output of the performed repetitions (ES range = 0.27 to 0.85). Therefore, even though Rahimi [13] reported that in the AC/CC genotypes the total number of repetitions was the same following the ingestion of caffeine and placebo, caffeine might have still enhanced the average velocity and power of these repetitions. We would suggest that future research in this area explores both the quality and quantity of the performed repetitions, to provide a more comprehensive assessment of possible effects of caffeine.

### Strengths and limitations

Some of the key strengths of this study are: (a) the standardization of testing conditions, including nutritional intake, physical activity, and the time of day at which the testing is conducted; (b) the inclusion of trained individuals as study participants; (c) a broad range of exercise performance variables that were assessed as outcomes; (d) assessment of performance across a wide-range of loads in the bench press exercise and both quantity and quality of repetitions, when examining muscle endurance as the outcome variable.

There are several potential limitations of this study that need to be acknowledged. First, due to the low number of individuals with the CC genotype, we combined the AC and CC genotypes into one group. This is fairly common in this line of research, as the number of individuals with the CC genotype in the population is suggested to be ~ 10% [9]. To get around 10 to 12 participants with the CC genotype a study would need to screen from 100 to 120 potential study participants. However, despite the fact this is a common practice, it could have confounded findings, as the effects of caffeine might not be uniform between individuals with the AC vs. CC genotype [10, 42]. In the current study, we could not test this further, because the number of individuals with the CC genotype was *n* = 2. Of note, the exclusion of these two participants from the analysis did not alter the study results.

The second limitation is related to the efficacy of blinding [24]. Previous research has established that correct supplement identification may impact the outcomes of a given exercise test and, therefore, bias the results. In the present study, around 50–60% of the participants were able to correctly identify the placebo and caffeine condition beyond random chance in the pre-exercise assessment. In the post-exercise assessment, this percentage generally stayed the same or slightly increased. We believe that the pre-exercise responses are of greater importance, given that the improvements during the testing session (or lack thereof) may influence the post-exercise responses. Tallis and colleagues [43] tested their participants in four conditions: (1) “told caffeine, given caffeine”; (2) “told caffeine, given placebo”; (3) “told placebo, given placebo”; and (4) “told placebo, given caffeine”. Equal improvements were found on both occasions when the participants indeed ingested caffeine (i.e., “told caffeine, given caffeine” and “told placebo, given caffeine” conditions), thus suggesting that this limitation of our study might not have greatly affected our findings.

## Conclusions

This study found that caffeine is acutely ergogenic for movement velocity, power output, and muscle endurance in resistance exercise, vertical jump height, and peak, mean, and minimum power in a Wingate test. These performance-enhancing effects were observed following the ingestion of using a moderate dose of caffeine (3 mg/kg), which resulted in minimal side effects. The comparisons of the effects of caffeine on exercise performance between individuals with the AA genotype and AC/CC genotypes found no significant differences.

## Data Availability

The datasets used and/or analyzed during the current study are available from the corresponding author on reasonable request.

## Abbreviations

1RM: One repetition maximum
ANOVA: Analysis of variance
CI: Confidence interval
CMJ: Countermovement jump
ES: Effect size
PAR-Q: Physical Activity Readiness Questionnaire
PCR: Polymerase chain reaction
